# A telemonitoring intervention design for patients with poorly controlled type 2 diabetes: protocol for a feasibility study

**DOI:** 10.1186/s40814-024-01509-0

**Published:** 2024-05-22

**Authors:** Sisse H. Laursen, Iben Engelbrecht Giese, Flemming W. Udsen, Ole K. Hejlesen, Pernille F. Barington, Morten Ohrt, Peter Vestergaard, Stine Hangaard

**Affiliations:** 1 Steno Diabetes Center North Denmark, Aalborg University Hospital, Aalborg, Denmark; 2 Department of Health Science and Technology, Aalborg University, Gistrup, Denmark; 3 Clinical Nursing Research Unit, Aalborg University Hospital, Aalborg, Denmark; 4 Department of Endocrinology, Aalborg University Hospital, Aalborg, Denmark; 5 Department of Clinical Medicine, Aalborg University, Aalborg, Denmark; 6 Nord-KAP, The Quality Unit for General Practice in the North Denmark Region, Aalborg, Denmark

**Keywords:** Blood glucose, Diabetes mellitus, Type 2, Telemedicine, Telehealth, Telemonitoring, Feasibility

## Abstract

**Background:**

Maintaining optimal glycemic control in type 2 diabetes (T2D) is difficult. Telemedicine has the potential to support people with poorly regulated T2D in the achievement of glycemic control, especially if the telemedicine solution includes a telemonitoring component. However, the ideal telemonitoring design for people with T2D remains unclear. Therefore, the aim of this feasibility study is to evaluate the feasibility of two telemonitoring designs for people with non-insulin-dependent T2D with a goal of identifying the optimal telemonitoring intervention for a planned future large-scale randomized controlled trial.

**Method:**

This 3-month randomized feasibility study will be conducted in four municipalities in North Denmark starting in January 2024. There will be 15 participants from each municipality. Two different telemonitoring intervention designs will be tested. One intervention will include self-monitoring of blood glucose (SMBG) combined with sleep and mental health monitoring. The second intervention will include an identical setup but with the addition of blood pressure and activity monitoring. Two municipalities will be allocated to one intervention design, whereas the other two municipalities will be allocated to the second intervention design. Qualitative interviews with participants and clinicians will be conducted to gain insight into their experiences with and acceptance of the intervention designs and trial procedures (e.g., blood sampling and questionnaires). In addition, sources of differences in direct intervention costs between the two alternative interventions will be investigated.

**Discussion:**

Telemonitoring has the potential to support people with diabetes in achieving glycemic control, but the existing evidence is inconsistent, and thus, the optimal design of interventions remains unclear. The results of this feasibility study are expected to produce relevant information about telemonitoring designs for people with T2D and help guide the design of future studies. A well-tested telemonitoring design is essential to ensure the quality of telemedicine initiatives, with goals of user acceptance and improved patient outcomes.

**Trial registration:**

ClinicalTrials.gov, ID: NCT06134934. Registered November 1, 2023. The feasibility trial has been approved (N-20230026) by the North Denmark Region Committee on Health Research Ethics (June 5, 2023).

**Supplementary Information:**

The online version contains supplementary material available at 10.1186/s40814-024-01509-0.

## Background

Diabetes represents a major health challenge worldwide. In 2017, it was estimated that 8.4% (451 million) of the adult global population had diabetes. The prevalence is expected to increase to approximately 9.9% (693 million) by 2045, for instance due to an increase in obesity and unhealthy diets [1–4]. Type 2 diabetes (T2D) accounts for approximately 90–95% of diabetes cases [5, 6].

To prevent and control diabetes-related complications, it is crucial to maintain glycemic control [7, 8]. However, optimal glycemic control is often difficult to maintain [7, 9], and less than 50% of people with diabetes reach their goal of glycemic control [10, 11]. The main challenge is that people with diabetes are highly responsible for disease management outside of hospital settings. The person with diabetes is required to perform complex care activities and make numerous daily decisions regarding self-management [9]. Therefore, alternative approaches in diabetes care are needed to support people with diabetes in achieving the desired treatment goals. In several studies, telemedicine has led to positive results in supporting people with diabetes [12–23]. Telemedicine solutions involve the transfer of information or data between a health care professional (HCP) and patients over a geographical distance and the provision of tailored feedback [24]. Telemedicine solutions vary [25, 26], ranging from simple short message service reminders to more complex telemonitoring solutions where the patient performs selected measurements at home and transfers their data to monitoring HCPs [16, 17, 20, 27–31]. Thus, telemedicine has a large potential to support people with diabetes in achieving glycemic control. Moreover, for those who are unable to travel to a health care clinic, telemedicine has the potential to increase access and provide better health outcomes [32].

In a comprehensive 2017 review, it was reported that telemedicine for people with diabetes is a safe way to provide support for self-care [33]. Several other evaluations of telemedicine solutions have shown varying results but with a positive trend considering glycemic control [15, 26, 34–37]. A review and meta-analysis by Faruque et al. from 2017 showed an improvement in glycated hemoglobin (HbA1c) in people with T2D who used telemedicine as a supplement to regular care [26]. Furthermore, in 2021 Hangaard et al. performed a systematic review, meta-analysis, and meta-regression focusing on T2D; they concluded that telemedicine may serve as a valuable supplement to usual care, especially if the solution includes a telemonitoring component, and that patients with poor glycemic control may benefit more from telemedicine than their well-regulated counterparts [16]. However, the ideal telemonitoring setup for T2D remains to be determined [16].

Telemonitoring interventions may have the potential to postpone the start time for insulin treatment and reduce the risk of diabetes-related complications in people with T2D if they focus on diabetes self-management education components. Such interventions could potentially provide the foundation for people with T2D to navigate the daily self-management and care activities related to diabetes [38, 39]. Diabetes self-management education components are the elements that facilitate skills, knowledge, and ability necessary for diabetes self-care [39]. Such components are the first step in T2D management and have been shown to improve health outcomes in people with diabetes [38, 40–42]. Different telemonitoring approaches focused on diabetes self-management education could be relevant in supporting people with non-insulin-dependent T2D. One essential approach is self-monitoring of blood glucose (SMBG), as it provides instant feedback on glycemic values rather than waiting for the next HbA1c [8]. Another relevant approach is monitoring sleep. Sleep disorders are prevalent in T2D and are associated with impaired glucose control and an increased risk of developing diabetes complications [43, 44]. Conversely, diabetes and its complications are associated with poor sleep quality, insomnia, and higher use of sleep medications [45, 46]. An increased awareness of proper sleep is important, as sufficient sleep can prevent diabetes progression [43, 44]. Thus, improvement of sleep could aid the treatment and course of T2D [44]. A third approach is monitoring of mental health, as people with diabetes are known to have more psychological problems compared to the general population associated with increased health care costs [47, 48]. In this regard, telemedicine specifically designed to help people with diabetes understand and better manage mental health symptoms has been shown to decrease anxiety, depression, and stress in people with diabetes [48]. A fourth relevant approach is monitoring blood pressure. Blood pressure levels are higher among people with T2D, and increased values are a well-established risk factor for cardiovascular events [49–51]. Thus, lowering blood pressure in people with T2D is associated with improved clinical outcomes and reduced mortality [51]. Finally, a fifth relevant telemonitoring approach is monitoring and follow-up on physical activity. Thus, studies show that physical activity and reducing sedentary behavior are essential for maintaining glycemic control in people with T2D [52, 53].

## Objectives

The main objective of this study is to evaluate the feasibility of different telemonitoring intervention designs for people with non-insulin-dependent T2D as a supplement to usual care (i.e., regular diabetes controls with the patient’s general practitioner) with the goal of identifying the most suitable design for a planned future large-scale randomized controlled trial. The study is designed to build on a delimited set of intervention components of presumed greatest importance to people with non-insulin-dependent T2D to help prioritize the components in the planned randomized controlled trial.

A secondary objective is to gain useful information for the design of the planned future randomized controlled trial. Apart from evaluating and identifying the most suitable intervention design, the study is expected to provide relevant information on the target group, sample size estimation, recruitment opportunities and challenges, suitable outcome measures, and follow-up rates [54]. Furthermore, the feasibility study is intended to help clarify whether the target group has specific wishes and requirements for a future telemedicine solution as well as whether there is any aspect of the group's social environment that requires special attention [55].

## Methods

This protocol paper is reported in accordance with an adjusted version of the SPIRIT2013 Statement: Defining standard protocol items for clinical trials [56]. The Standard Protocol Items: Recommendations for Interventional Trials (SPIRIT) checklist is available in Additional file 1. The adjustments made include the suggestions by Lehana Thabane & Gillian Lancaster in “A guide to the reporting of protocols of pilot and feasibility trials” [57], a guide that includes items from the CONSORT 2010 statement: extension to randomized pilot and feasibility trials [58].

### Study design and setting

**Fig. 1:**
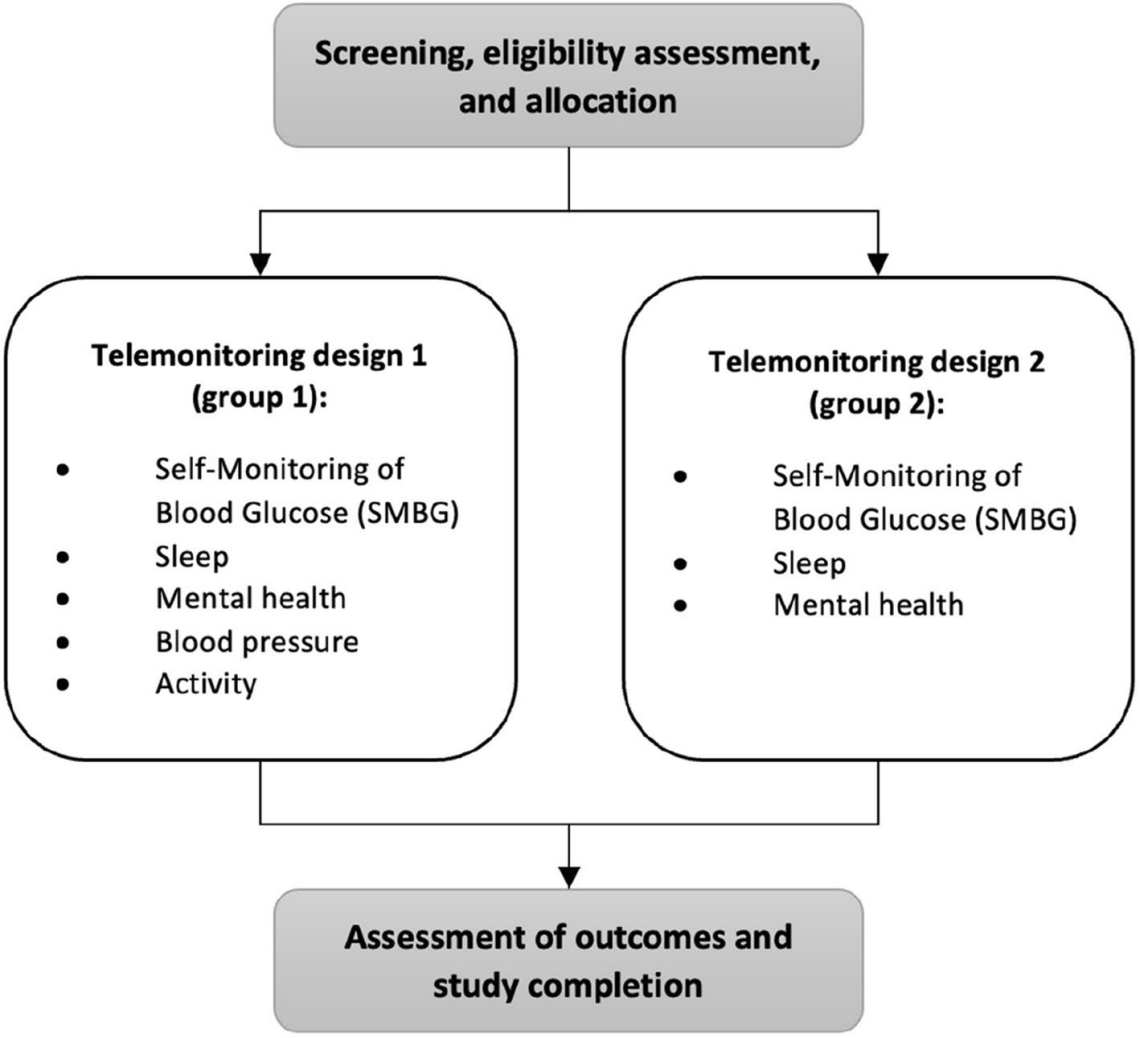
Feasibility study design

The trial is a feasibility study with a trial period of 3 months. The trial will be conducted as a randomized cluster study in four municipalities in North Denmark (Hjørring, Morsø, Jammerbugt, and Rebild). Two different telemonitoring designs will be tested to identify the most suitable telemonitoring intervention for a future randomized controlled trial on a large scale. Two municipalities will test one intervention design (group 1), while the other two municipalities will test the second intervention design (group 2). The overall trial design is illustrated in Fig. 1.

The municipalities will be randomized using a drawing of envelopes approach. The drawing will be performed in groups based on the setting of the respective municipalities to ensure that each intervention design will be tested in a health care center as well as in a home health care setting.

All participants will be provided with a telemonitoring account (OpenTeleHealth North account), a blood glucose monitoring kit, and a tablet (unless the participant prefers to use his or her own tablet or smartphone) at trial initiation. The participants will monitor sleep and mental health status through questions implemented in the telemonitoring system. Sleep will be monitored using sleep item questions from a Danish patient-reported outcome (PRO) questionnaire for T2D, while mental health will be monitored through the World Health Organization Five Well-being Index (WHO-5) questionnaire. In addition, the participants in group 1 will be provided with a blood pressure monitor and an activity tracker.

All participants will be trained in using the provided technologies at trial initiation. They will use the distributed devices continuously at home to collect, log, and transfer data to the municipality nurse for the entire trial duration. The municipality nurses will monitor the participants’ data weekly using the telemonitoring system. They will contact the participants by phone, video, or text message at least every other week during the first 6 weeks of the trial. For the final 6 weeks, the frequency of contacts will be tailored based on the needs of the individual participant. Hence, participants may be contacted more frequently than every other week if it is considered relevant by the monitoring municipality nurse. All calls to participants will be recorded in their respective electronic care journals as a part of mandatory registration for the municipality nurse. Participants will be informed that they remain responsible for monitoring and managing their blood glucose levels despite telemonitoring. Furthermore, since the intervention should be seen as a supplement to usual care, the participants are informed that they should continue usual care (diabetes-related visits and controls) with their general practitioner during the trial.

At the end of the trial, participants from both intervention groups will have a final visit with the monitoring municipality nurse. The visit can take place either at the local municipality trial site or at the participant’s home.

Due to the nature of the trial, it will not be possible to blind participants, HCPs, or researchers.

### Outcomes and data collection

**Fig. 2:**
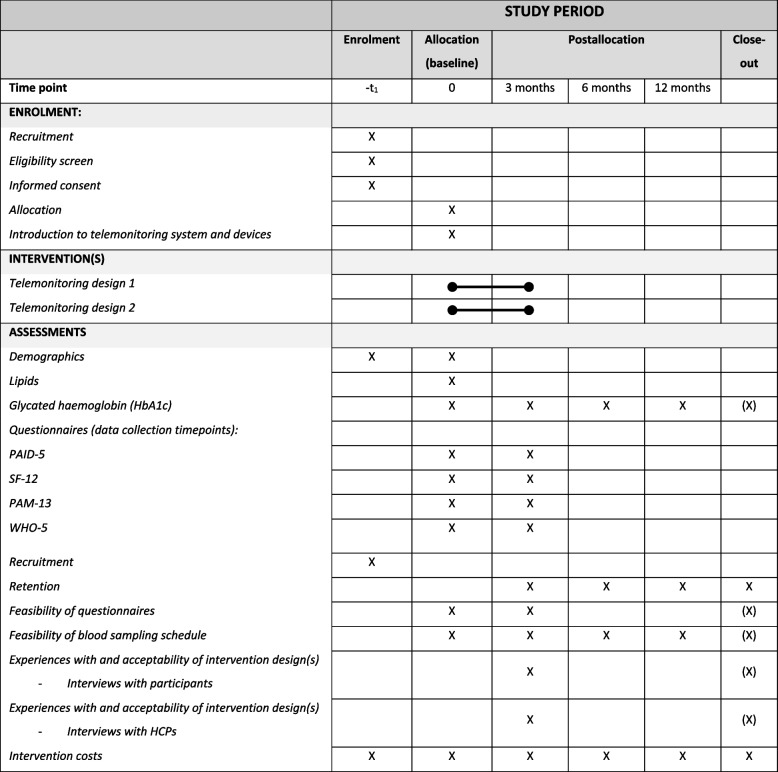
SPIRIT figure. Participant timeline with schedule of enrolment, intervention(s), and assessment time points for both intervention groups

The outcomes are used to explore the two different intervention designs to identify the best possible design for a future randomized controlled trial and not to define the statistical or clinical effectiveness of the two interventions. The SPIRIT figure (Fig. 2) provides an overview of the time schedule for enrolment, interventions, and assessments as anticipated in the feasibility trial.

#### Demographics

Patient demographics will be obtained at trial initiation, including age (civil registration number), sex, height, weight, blood pressure, civil status, educational level, duration of diabetes, diabetes complications, concomitant medications, concomitant illness, and health-related habits. In addition, baseline HbA1c and lipid values will be used to characterize the participants (blood sampling procedures are explained in the “ Feasibility of the blood sampling schedule” section).

#### Recruitment process

The recruitment assessment will include the following:

- Number and proportion of people agreeing to receive a participant information letter about the trial
- Number and proportion of eligible participants who agree to participate
- Potential inequalities regarding recruitment feasibility will be assessed by comparing demographic data on age, sex, ethnicity, educational level, municipality, setting (health care center versus home health care setting), and HbA1c (baseline)

#### Retention

The retention assessment will include the following:

- Number and proportion of participants withdrawing from the trial
- Timepoint(s) for withdrawal
- Reasons for discontinuation of the trial
- Potential inequalities regarding retention assessed by comparing demographic data on age, sex, ethnicity, educational level, municipality, setting (health care center versus home health care setting), and HbA1c (baseline)

#### Feasibility of questionnaires

Another outcome is to evaluate the use of selected questionnaires in the intervention designs based on response rates. This will be done to assess their suitability for the patient group and the future large-scale randomized controlled trial. Therefore, the participants will be encouraged to answer the following four questionnaires at baseline and at the end of the trial approximately three months after inclusion:

1. Diabetes-related quality of life: The Problem Areas in Diabetes Questionnaire (PAID5)
2. Quality of Life: The Short Form 12 Questionnaire (SF-12v2)
3. Well-being: The World Health Organization Five Well-being Index (WHO-5)
4. Knowledge, skills, and confidence in managing health: The Patient Activation Measure questionnaire (PAM)

#### Feasibility of the blood sampling schedule

HbA1c will be collected at baseline and 3, 6, and 12 months after inclusion. In addition, lipids will be collected at baseline together with HbA1c. Blood samples will be drawn to explore the following:

- If there are any analysis challenges or uncertainties
- Adherence among participants to the blood sampling schedule: number and proportion of the participants who completes the blood sampling at baseline, 3 months, 6 months, and 12 months

All blood samples will be drawn by staff at the participant’s general practitioner. The samples will be analyzed as soon as possible after extraction at Aalborg University Hospital or at the North Denmark Regional Hospital but no later than 5 days after extraction, after which the samples will be destroyed. Data on HbA1c at 6 and 12 months after inclusion will be based on blood samples collected as part of regular clinical practice.

#### Experiences with and acceptability of intervention design(s)

Following the 3-month intervention period, individual semi-structured qualitative interviews will be conducted with selected participants and HCPs to gain deeper insight into the participants’ and HCPs’ experiences and acceptability with the two different telemonitoring intervention designs (cf. the “ Study design and setting” section) and the trial procedures. The interviews will, for example, include questions concerning the following information:

- How they experience and feel about the intervention and the telemonitoring equipment and design
- How they assess the burden of being involved in the intervention
- If they have any ethical concerns about participating in the intervention

In addition, the interviews will cover the following information:

- Recruitment opportunities and challenges
- Perspectives on retention problems
- Perspectives on the use of selected questionnaires in the intervention designs and the use of the PRO (sleep items) and the WHO-5 questions as part of the telemonitoring intervention during the trial (cf. section “ Study design and setting”)
- If the planned timepoints for blood sampling are suitable and meaningful for the future large-scale randomized controlled trial considering potential challenges in relation to social factors, clinical workflows, processes, etc.

#### Intervention costs

Using interviews with HCPs and administrative personnel, a particular focus throughout the feasibility study is to investigate sources of direct and indirect intervention costs for the two alternative intervention designs across the region, the included municipalities, and general practitioners. Study-induced costs will be excluded (e.g., time spent distributing study questionnaires and other activities not included when implementing the potential interventions in routine practice). Potential direct resource categories are in principle uncertain but could include equipment and time spent on monitoring, time spent training patients in using the equipment, additional training for community nurses, and additional time spent by general practitioners in running the offer. Indirect costs could include software licensing, technical support, swapping defect equipment, IT maintenance, server allocation space, etc.

#### Eligibility criteria

The inclusion criteria for participating in the feasibility study will be as follows:

- Adults ≥ 18 years
- Poorly controlled T2D, i.e., HbA1c > 58 mmol/mol
- Diagnosis of T2D for at least 12 months
- General practitioner responsible for diabetes treatment
- Residence in Hjørring, Morsø, Jammerbugt, or Rebild municipality
- Ability and willingness to use a smartphone/tablet along with the other devices to be used in the trial
- Signed informed consent
- Ability to understand and read Danish

The exclusion criteria will be as follows:

- Pregnancy or breastfeeding
- Insulin treatment
- Prednisolone treatment
- Severe diabetes complications such as severe neuropathy or nephropathy (dialysis treatment)
- Participation in diabetes rehabilitation courses and in other intervention trials
- Terms that, in the opinion of the sub-investigator or investigator, render the participant unfit to conduct the trial, including lack of understanding of the trial or lack of physical or cognitive ability to participate.

### Sample size

Power calculation will not be conducted, as hypothesis testing is inappropriate in feasibility studies and therefore not an objective of this trial [54]. Instead, the sample size will be based on recommendations in the literature. A general rule is a sample size of 30 patients or greater in pilot studies [59]. Furthermore, 12 [60] to 50 [61] participants per arm have been suggested. Based on these recommendations, 60 participants will be included in the feasibility study, with 30 participants undergoing each intervention design.

### Recruitment and ethical considerations

Eligible people with non-insulin-dependent T2D will be recruited through general practitioners (or HCPs from the municipalities can send an inquiry to general practice) in the four municipalities in connection with diabetes consultations. The general practitioners will briefly inform the participant about the trial, hand out a participant information letter to interested patients, and refer them electronically to the municipality project nurses for potential inclusion. The potential participants will subsequently be called in for an information meeting with the possibility of a companion. This will be described in the participant information letter, which will also explain the purpose and design of the trial. During this meeting, the potential participant will be given in-depth information about the trial and will have the opportunity to ask questions. Moreover, attempts will be made to determine whether the potential participant is motivated and suited to participate in the telemonitoring trial. The information meeting will take place in a closed room at one of the municipality sites or in the participant’s home, where the conversation can take place undisturbed. The information meeting will be conducted by a municipality nurse (authorized by the primary investigator to perform the task) from the project team with the necessary professional knowledge. During the meeting, the participant will be made aware of her or his right to a reflection period of at least 24 h prior to giving informed consent and that the consent can be withdrawn at any time and without justification. Only when informed consent has been obtained with the signature of both the participant and the municipality nurse will the trial begin.

The recruitment of participants for the interviews will be conducted continuously due to ongoing inclusion of eligible participants throughout the whole trail period. Toward the end of the three months trail period, selected participants will be contacted by one of the researchers to receive information about the interview and asked about interest in possible participation. To ensure broad perspectives, a maximum variation sampling strategy will be sought to ensure that the interviews cover perspectives from both telemonitoring designs and both settings (i.e., health care center and home health care setting). Moreover, attempts will be made to include participants of varying genders and age groups.

Recruitment of HCPs for the interviews will be conducted towards the end of the entire trail period. It will be aspired to recruit one HCP for each municipality to ensure a broad perspective across municipality nurses. However, the recruitment process will depend upon organizational and logistical feasibility such as resources and work schedules.

The trial will be terminated in the event of any serious adverse events related to the trial as considered by the primary investigator. Furthermore, the trial will be stopped for the individual participant if severe hypoglycemia (low blood glucose; severe defined as levels ≤ 54 mg/dL), ketoacidosis (when ketone acids are build up to potentially dangerous levels in the body), or severe hyperglycemia (high blood glucose; severe defined as levels ≥ 180 mg/dl) is recorded and determined by the primary investigator to be related to the trial. However, the risk of such events is low since participants on insulin therapy will be excluded. If the trial is stopped for the individual participant or if a participant requests to withdraw from the trial, a subsequent final meeting with a municipality nurse is offered. At this visit, any questions from the participant will be answered.

The trial will be carried out in accordance with the Helsinki Declaration [62] and the principles of good clinical practice (GCP) [63]. Furthermore, the North Denmark Region Committee on Health Research Ethics approved the trial (Project ID: N-20230026).

### Analysis

Descriptive statistics will be used to present baseline demographics. Continuous data will be summarized using the mean and standard deviation, while categorical data will be presented as percentages.

The data collected from the qualitative interviews will be analyzed through inductive thematic analysis to identify central themes.

The extent of missing data from the participants throughout the trial period, response rate for the questionnaires, and number of completed blood samplings will be collected and presented using descriptive statistics. The data will be used to evaluate acceptability and adherence among the participants to the two intervention designs.

The intervention design for a future randomized controlled trial will be determined by:

1. Comparison of the quantitative results (recruitment, retention, and intervention costs) derived from the two intervention designs. If there is no notable difference in the quantitative results between the two intervention designs, the design with the least number of components will immediately be preferred to ensure the most favorable solution for a larger population
2. An overall assessment, incorporating the quantitative results and findings from the qualitative interviews (with participants and HCPs), through dialogue between the researchers and involved personnel from the North Denmark Region, municipalities, and general practitioners

As already stated, no sample size calculation will be performed since the present study is a feasibility trial. However, the results from the trial are expected to provide useful information on sample size calculation for the future randomized controlled trial regarding drop-out estimation.

### Plans for data quality and security

Various measures will be taken to promote data quality and security. The data will be entered and stored in the secure web application the Research Electronic Data Capture (REDCap) system [64] using double data entry to ensure the integrity of the captured data. This is especially important, as a large proportion of the data will be initially collected in paper form and then subsequently entered into the REDCap system. Furthermore, range checks for the data values will be used to validate data if it is considered appropriate that the data fall within a certain range. Finally, secure drives will be used if the researchers need to work on the dataset outside of REDCap.

## Discussion

The feasibility study aims to develop and test two telemonitoring designs for people with non-insulin-dependent T2D with the goal of identifying the most suitable telemonitoring intervention for a planned future large-scale randomized controlled trial. The study is innovative from different perspectives. First, the study will be carried out in North Denmark, a region where no telemedicine solution for diabetes exists despite the positive effects that have been shown in previously mentioned studies [16, 26] and despite an existing telehealth organization in the region (TeleCare North) [65]. Thus, there is a need to develop and test a telemonitoring design for T2D with a goal of future implementation in the North Denmark Region. Furthermore, the inclusion of four municipalities in the study will help increase the trial's external validity [66]. Evaluating a telemonitoring solution for people with non-insulin-dependent T2D is expected to produce relevant information about telemonitoring designs for the patient group and may help guide the design process of future studies, including the planned future randomized controlled trial in large scale. A well-adapted and well-tested telemonitoring design is essential to ensure the quality of telemedicine initiatives in general with a view to better user acceptance and patient outcomes [55].

The feasibility study will also be associated with some limitations. The study will not enable evaluation of the intervention effects or specific subgroup effects nor will it evaluate the effect compared to usual care as intended in the planned future randomized trial. However, the design of feasibility studies is common as a first step in the development process and evaluation of new and future interventions [54, 59]. Another limitation is the opt-out of other intervention combinations. In this study, only two intervention combinations will be tested and compared. Other set-ups could be relevant. However, the feasibility study has been carefully designed to build on a delimited set of intervention components of presumed greatest importance to people with non-insulin-dependent T2D. Furthermore, several HCPs with expertise in diabetes and a user advisory council with potential end-users were involved in the design process. Utilizing a few carefully selected intervention components will hopefully help prioritize future studies and telemonitoring designs. It could be relevant, however, to test other intervention components and combinations in future studies.

### Supplementary Information

**Additional file 1. **SPIRIT 2013 Checklist: Recommended items to address in a clinical trial protocol and related documents.

## Data Availability

Not applicable.

## Abbreviations

HCP: Health care professional
GCP: Good clinical practice
GLM: Generalized linear model
HbA1c: Glycated hemoglobin
PAID-5: Problem Areas in Diabetes Questionnaire
PRO: Patient-reported outcome
PAM-13: The Patient Activation Measure questionnaire
REDCap: The Research Electronic Data Capture system
SF-12: The Short Form 12 Questionnaire
SMBG: Self-monitoring of blood glucose
SPIRIT: Standard Protocol Items: Recommendations for Interventional Trials
WHO-5: World Health Organization Five Well-being Index
T2D: Type 2 diabetes

## References

[1] Cho NH, Shaw JE, Karuranga S, Huang Y, da Rocha Fernandes JD, Ohlrogge AW (2018). IDF Diabetes Atlas: global estimates of diabetes prevalence for 2017 and projections for 2045. Diabetes Res Clin Pract.

[2] Ng M, Fleming T, Robinson M, Thomson B, Graetz N, Margono C (2014). Global, regional, and national prevalence of overweight and obesity in children and adults during 1980–2013: a systematic analysis for the Global Burden of Disease Study 2013. Lancet.

[3] Hu FB (2011). Globalization of diabetes: the role of diet, lifestyle, and genes. Diabetes Care.

[4] World Health Organization (2021). Diabetes.

[5] Xu G, Liu B, Sun Y, Du Y, Snetselaar LG, Hu FB (2018). Prevalence of diagnosed type 1 and type 2 diabetes among US adults in 2016 and 2017: population based study. BMJ.

[6] International Diabetes Federation (2017). IDF Diabetes Atlas.

[7] Bonora E, DeFronzo RA (2018). Diabetes complications, comorbidities and related disorders.

[8] Frías-Ordoñez JS, Pérez-Gualdrón CE (2019). Self-monitoring of blood glucose as control tool in the different management contexts for type 2 diabetes mellitus. What is its current role in non-insulin users?. Rev Fac Med.

[9] Powers MA, Bardsley J, Cypress M, Duker P, Funnell MM, Fischl AH (2015). Diabetes self-management education and support in type 2 diabetes: a joint position statement of the American Diabetes Association, the American Association of Diabetes Educators, and the Academy of Nutrition and Dietetics. Diabetes Care.

[10] Carls G, Huynh J, Tuttle E, Yee J, Edelman SV (2017). Achievement of glycated hemoglobin goals in the US remains unchanged through 2014. Diabetes Ther.

[11] National Committee for Quality Assurance (2015). The state of health care quality report.

[12] Aguiar PM, da Silva CHP, Chiann C, Dórea EL, Lyra DP, Storpirtis S (2018). Pharmacist–physician collaborative care model for patients with uncontrolled type 2 diabetes in Brazil: results from a randomized controlled trial. J Eval Clin Pract.

[13] Anderson RM, Funnell MM, Aikens JE, Krein SL, Fitzgerald JT, Nwankwo R (2009). Evaluating the efficacy of an empowerment-based self-management consultant intervention: results of a two-year randomized controlled trial. Educ Ther du Patient.

[14] Hu Y, Wen X, Wang F, Yang D, Liu S, Li P (2019). Effect of telemedicine intervention on hypoglycaemia in diabetes patients: a systematic review and meta-analysis of randomised controlled trials. J Telemed Telecare.

[15] Su D, Zhou J, Kelley MS, Michaud TL, Siahpush M, Kim J, Wilson F, Stimpson JP (2016). Does telemedicine improve treatment outcomes for diabetes? A meta-analysis of results from 55 randomized controlled trials. Diabetes Res Clin Pract.

[16] Hangaard S, Laursen SH, Andersen JD, Kronborg T, Vestergaard P, Hejlesen O (2021). The effectiveness of telemedicine solutions for the management of type 2 diabetes: a systematic review, meta-analysis, and meta-regression. J Diabetes Sci Technol.

[17] Avdal EÜ, Kizilci S, Demirel N (2011). The effects of web-based diabetes education on diabetes care results a randomized control study. Comput Informatics Nurs.

[18] Bogner H (2012). Diabetes mellitus and depression treatment to improve medication adherence. Ann Fam Med.

[19] Chen HS, Wu TE, Jap TS, Lin SH, Hsiao LC, Lin HD (2008). Improvement of glycaemia control in subjects with type 2 diabetes by self-monitoring of blood glucose: comparison of two management programs adjusting bedtime insulin dosage. Diabetes Obes Metab.

[20] Fortmann AL, Gallo LC, Garcia MI, Taleb M, Euyoque JA, Clark T (2017). Dulce digital: an mHealth SMS based intervention improves glycemic control in hispanics with type 2 diabetes. Diabetes Care.

[21] Holbrook A, Thabane L, Keshavjee K, Dolovich L, Bernstein B, Chan D (2009). Individualized electronic decision support and reminders to improve diabetes care in the community: COMPETE II randomized trial. C Can Med Assoc J.

[22] Ralston JD, Hirsch IB, Hoath J, Mullen M, Cheadle A, Goldberg HI (2009). Web-based collaborative care for type 2 diabetes a pilot randomized trial. Diabetes Care.

[23] Rasmussen OW, Lauszus FF, Loekke M (2016). Telemedicine compared with standard care in type 2 diabetes mellitus: a randomized trial in an outpatient clinic. J Telemed Telecare.

[24] McLean S, Nurmatov U, Liu JL, Pagliari C, Car J, Sheikh A (2012). Telehealthcare for chronic obstructive pulmonary disease: Cochrane Review and meta-analysis. Br J Gen Pract.

[25] Kaufman N, Ferrin C, Sugrue D (2019). Using digital health technology to prevent and treat diabetes. Diabetes Technol Ther.

[26] Faruque LI, Wiebe N, Ehteshami-Afshar A, Liu Y, Dianati-Maleki N, Hemmelgarn BR (2017). Effect of telemedicine on glycated hemoglobin in diabetes: a systematic review and meta-analysis of randomized trials. CMAJ.

[27] Crico C, Renzi C, Graf N, Buyx A, Kondylakis H, Koumakis L (2018). mHealth and telemedicine apps: in search of a common regulation. Ecancermedicalscience.

[28] Mahar JH, Rosencrance GJ, Rasmussen PA (2018). Telemedicine: past, present, and future. Cleve Clin J Med.

[29] Capozza K, Woolsey S, Georgsson M, Black J, Bello N, Lence C (2015). Going mobile with diabetes support: a randomized study of a text message-based personalized behavioral intervention for type 2 diabetes self-care. Diabetes Spectr.

[30] Carter EL, Nunlee-Bland G, Callender C (2011). A patient-centric, provider-assisted diabetes telehealth self-management intervention for urban minorities. Perspect Heal Inf Manag.

[31] Cho JH, Kim H, Yoo SH, Jung CH, Lee WJ, Park CY (2017). An Internet-based health gateway device for interactive communication and automatic data uploading: clinical efficacy for type 2 diabetes in a multi-centre trial. J Telemed Telecare.

[32] Shah TK, Tariq T, Phillips R, Davison S, Hoare A, Hasan SS (2018). Health care for all: effective, community supported, healthcare with innovative use of telemedicine technology. J Pharm Policy Pract.

[33] Hanlon P, Daines L, Campbell C, McKinstry B, Weller D, Pinnock H (2017). Telehealth interventions to support self-management of long-term conditions: a systematic metareview of diabetes, heart failure, asthma, chronic obstructive pulmonary disease, and cancer. J Med Internet Res.

[34] Baron J, McBain H, Newman S (2012). The impact of mobile monitoring technologies on glycosylated hemoglobin in diabetes: a systematic review. J Diabetes Sci Technol.

[35] Holtz B, Lauckner C (2012). Diabetes management via mobile phones: a systematic review. Telemed e-Health.

[36] Jaana M, Paré G (2007). Home telemonitoring of patients with diabetes: a systematic assessment of observed effects. J Eval Clin Pract.

[37] Greenwood DA, Young HM, Quinn CC (2014). Telehealth remote monitoring systematic review: structured self-monitoring of blood glucose and impact on A1C. J Diabetes Sci Technol.

[38] Steinsbekk A, Rygg L, Lisulo M, Rise MB, Fretheim A (2012). Group based diabetes self-management education compared to routine treatment for people with type 2 diabetes mellitus. A systematic review with meta-analysis. BMC Heal Serv Res.

[39] Powers MA, Bardsley J, Cypress M, Duker P, Funnell MM, Fischl AH (2016). Diabetes self-management education and support in type 2 diabetes: a joint position statement of the American Diabetes Association, the American Association of Diabetes Educators, and the Academy of Nutrition and Dietetics. Clin Diabetes.

[40] Weaver RG, Hemmelgarn BR, Rabi DM, Sargious PM, Edwards AL, Manns BJ (2014). Educational and psychological issues association between participation in a brief diabetes education programme and glycaemic control in adults with newly diagnosed diabetes. Diabet Med.

[41] Duncan I, Birkmeyer C, Coughlin S, Li QE, Sherr D, Boren S (2009). Assessing the value of diabetes education. Diabetes Educ.

[42] Fan L, Sidani S (2009). Effectiveness of diabetes self-management education intervention elements: a meta-analysis. Can J Diabetes.

[43] Schipper SBJ, Van VMM, Van Der WYD, Knutson KL, Elders PJM (2021). Sleep disorders in people with type 2 diabetes and associated health outcomes: a review of the literature. Diabetologia.

[44] Tan X, van Egmond L, Chapman CD, Cedernaes J, Benedict C (2018). Aiding sleep in type 2 diabetes: therapeutic considerations. Lancet Diabetes Endocrinol.

[45] Skomro RP, Ludwig S, Salamon E, Kryger MH (2001). Sleep complaints and restless legs syndrome in adult type 2 diabetics. Sleep Meidicine.

[46] American Diabetes Association (2017). Comprehensive medical evaluation and assessment of comorbidities. Diabetes Care.

[47] Mochari-Greenberger H, Vue L, Luka A, Peters A, Pande RL (2016). A tele-behavioral health intervention to reduce depression, anxiety, and stress and improve diabetes self-management. Telemed e-Health.

[48] Egede LE, Gebregziabher M (2015). Differential impact of mental health multimorbidity on healthcare costs in diabetes. Am J Manag Care.

[49] Eguchi K, Pickering TG, Hoshide S, Ishikawa J, Ishikawa S, Schwartz JE (2008). Ambulatory blood pressure is a better marker than clinic blood pressure in predicting cardiovascular events in patients with/without type 2 diabetes. Am J Hypertens.

[50] Petrie JR, Guzik TJ, Touyz RM (2018). Diabetes, hypertension, and cardiovascular disease: clinical insights and vascular mechanisms. Can J Cardiol.

[51] Emdin CA, Rahimi K, Neal B, Callender T, Perkovic V, Patel A (2015). Blood pressure lowering in type 2 diabetes: a systematic review and meta-analysis. JAMA.

[52] Howland C, Wakefield B (2021). Assessing telehealth interventions for physical activity and sedentary behavior self - management in adults with type 2 diabetes mellitus: an integrative review. Res Nurs Heal.

[53] Van Dijk JW, Manders RJF, Tummers K, Bonomi AG, Stehouwer CDA, Hartgens F (2012). Both resistance- and endurance-type exercise reduce the prevalence of hyperglycaemia in individuals with impaired glucose tolerance and in insulin-treated and non-insulin-treated type 2 diabetic patients. Diabetologia.

[54] Arain M, Campbell MJ, Cooper CL, Lancaster G (2010). What is a pilot or feasibility study?. BMC Med Res Methodol.

[55] Harst L, Lantzsch H, Scheibe M (2019). Theories predicting end-user acceptance of telemedicine use: systematic review. J Med Internet Res.

[56] Chan A-W, Tetzlaff JM, Altman DG, Laupacis A, Gøtzsche PC, Krleža-Jerić K, Hróbjartsson A, Mann H, Dickersin K, Berlin J, Doré C, Parulekar W, Summerskill W, Groves T, Schulz K, Sox H, Rockhold FW, Rennie D, Moher D. SPIRIT 2013 Statement: Defining standard protocol items for clinical trials. Ann Intern Med. 2013;158:200–7. 10.7326/0003-4819-158-3-201302050-00583 PMC5114123 23295957

[57] Thabane L, Lancaster G (2019). A guide to the reporting of protocols of pilot and feasibility trials. Pilot Feasibility Stud.

[58] Eldridge SM, Chan CL, Campbell MJ, Bond CM, Hopewell S, Thabane L (2016). CONSORT 2010 statement: extension to randomised pilot and feasibility trials. BMJ.

[59] Lancaster GA, Dodd S, Williamson PR (2004). Design and analysis of pilot studies: recommendations for good practice. J Eval Clin Pract.

[60] Julious SA (2005). Sample size of 12 per group rule of thumb for a pilot study. Pharm Stat.

[61] Sim J, Lewis M (2012). The size of a pilot study for a clinical trial should be calculated in relation to considerations of precision and efficiency. J Clin Epidemiol.

[62] World Medical Association (2013). World Medical Association Declaration of Helsinki: ethical principles for medical research involving human subjects. JAMA.

[63] EUR-Lex - Access to European Union law. Available from: https://eur-lex.europa.eu/legal-content/EN/TXT/?uri=CELEX%3A32016R0679&qid=1686571590544. Cited 2023 Jun 12

[64] REDCap - Research Electronic Data Capture. Available from: https://www.project-redcap.org/. Cited 2023 Jun 12

[65] Telecare North. Large-scale telemedicine. 2013. Available from: https://rn.dk/sundhed/til-sundhedsfaglige-og-samarbejdspartnere/telecare-nord/telemedicin-kol/nordjyske-erfaringer-med-telemedicin/-/media/Rn_dk/Sundhed/Til-sundhedsfaglige-og-samarbejdspartnere/TelecareNord/Telemedicin-til-borgere-med-KOL/KOL-projekt/Mer. Cited 2023 Jun 12

[66] Findley MG, Kikuta K, Denly M (2021). External validity. Annu Rev Polit Sci.

